# PINGing Sunshine: A Review of the Evidence for Adding Non‐Filtering Photoprotective Ingredients to Sunscreens

**DOI:** 10.1111/phpp.70062

**Published:** 2025-10-28

**Authors:** Jean Krutmann, Anthony Brown, Thierry Passeron, Corinne Granger, Yolanda Gilaberte, Carles Trullas, Jaime Piquero‐Casals, Giovanni Leone, Sergio Schalka, Henry W. Lim

**Affiliations:** ^1^ IUF—Leibniz Research Institute for Environmental Medicine Duesseldorf Germany; ^2^ Innovation and Development, ISDIN Barcelona Spain; ^3^ Department of Dermatology Centre Hospitalier Universitaire de Nice, Côte D'azur University Nice France; ^4^ INSERM U1065 Centre Méditerranéen de Médecine Moléculaire (C3M), Côte D'azur University Nice France; ^5^ Stella Polaris Europe SAS Paris France; ^6^ Miguel Servet University Hospital, University of Zaragoza Zaragoza Spain; ^7^ Dermik Clinica Dermatologica Multidisciplinar Barcelona Spain; ^8^ Photodermatology Unit, Israelite Hospital Roma Italy; ^9^ Medcin Skin Research Center and Biochemistry Department Chemistry Institute of São Paulo University São Paulo Brazil; ^10^ Department of Dermatology Henry Ford Health Detroit Michigan USA

**Keywords:** antioxidants, DNA repair, infrared and visible light protection, nicotinamide, non‐filtering ingredients, oxidative stress, photolyase, photoprotection, skin photoaging, sunscreens

## Abstract

**Background:**

Photoprotective INGredients (PINGs) are non‐filtering agents that enhance the skin's intrinsic defenses against solar radiation. Acting through antioxidant, DNA repair, immunomodulatory, anti‐inflammatory, and pigmentation‐regulating mechanisms, PINGs may prevent or repair photodamage. When incorporated into sunscreens, they offer protection beyond ultraviolet (UV) filters. This strategy of biological photoprotection could address key limitations of traditional sunscreens and reduce dependence on high UV filter concentrations.

**Methods:**

We conducted a focused literature review based on our prior evidence‐based classification of over 1700 topical PINGs. We selected ingredients with the strongest clinical and mechanistic support and assessed their biological activity, formulation compatibility, and relevance to key endpoints such as erythema, pigmentation, photoaging, and immunosuppression.

**Results:**

Top‐ranked PINGs, including L‐ascorbic acid, tocopherol, photolyase, and nicotinamide, demonstrated efficacy across multiple photodamage endpoints. Antioxidants like L‐ascorbic acid and tocopherol enhanced protection against UVR and IR‐A‐induced oxidative stress. DNA repair enzymes, such as photolyase, reduced cyclobutane pyrimidine dimer formation and supported immune function. Nicotinamide improved DNA repair and prevented UV‐induced immunosuppression. Pigmentation modulators such as p‐coumaric acid and isobutylamido thiazolyl resorcinol showed benefits in darker phototypes.

**Conclusions:**

Fewer than 2% of candidate PINGs are clinically validated, and only 18 are approved for use in sunscreens. Protection against visible and infrared radiation remains largely underexplored. Standardized testing and additional clinical trials are needed to advance PINGs as effective components of next‐generation sunscreens.

## 1 Introduction

Regular sunscreen use remains one of the most effective ways to protect skin from sun‐induced damage. Sunscreens combine organic and inorganic filters to prevent ultraviolet radiation (UVR) from penetrating the skin. Organic filters act by absorption, while inorganic filters primarily absorb UVR and additionally contribute to scattering [1]. Most sunscreens are formulated to offer broad‐spectrum protection by combining UVB and UVA filters, and when properly applied, they significantly reduce the risk of sunburn, skin cancers, hyperpigmentation, photoaging, and photodermatoses [2]. In practice, however, sunscreens have several intrinsic limitations that compromise their effectiveness. Firstly, under real conditions of use, most users fail to apply and reapply enough sunscreen to the skin, thereby reducing the level of protection afforded by it in outdoor conditions [3]. Secondly, most currently available filters fail to protect against long wavelength UVA rays and non‐UVR, such as visible (VL) and infrared (IR) light, which also contribute to skin damage [4]. There are also increasing concerns about the impact of sunscreens on the aquatic environment that have led to a ban on the use of some UV filters in certain parts of the world [5]. Finally, consumers are increasingly demanding more natural, multifunctional products [6]. As a result, interest has grown in non‐filtering photoprotective ingredients, collectively referred to as photoprotective INGredients (PINGs), a term we previously introduced to describe this class of substances [7]. PINGs work by enhancing the skin's intrinsic defenses through antioxidant, DNA repair, immunomodulatory, pigmentation‐regulating, and anti‐inflammatory effects. PINGs may also offer anti‐aging and skin‐brightening benefits, broadening the appeal of sunscreens. Despite the growing number of photoprotective ingredient candidates, few have been subjected to rigorous, systematic evaluation. To address this gap, we previously conducted an evidence‐based analysis of the efficacy of PINGs used in combination with sunscreens [7]. In that study, we developed a structured scoring system to assess both the strength and quality of the scientific evidence supporting each ingredient. This framework allowed for a consistent and transparent appraisal of efficacy across a broad range of compounds.

**FIGURE 1:**
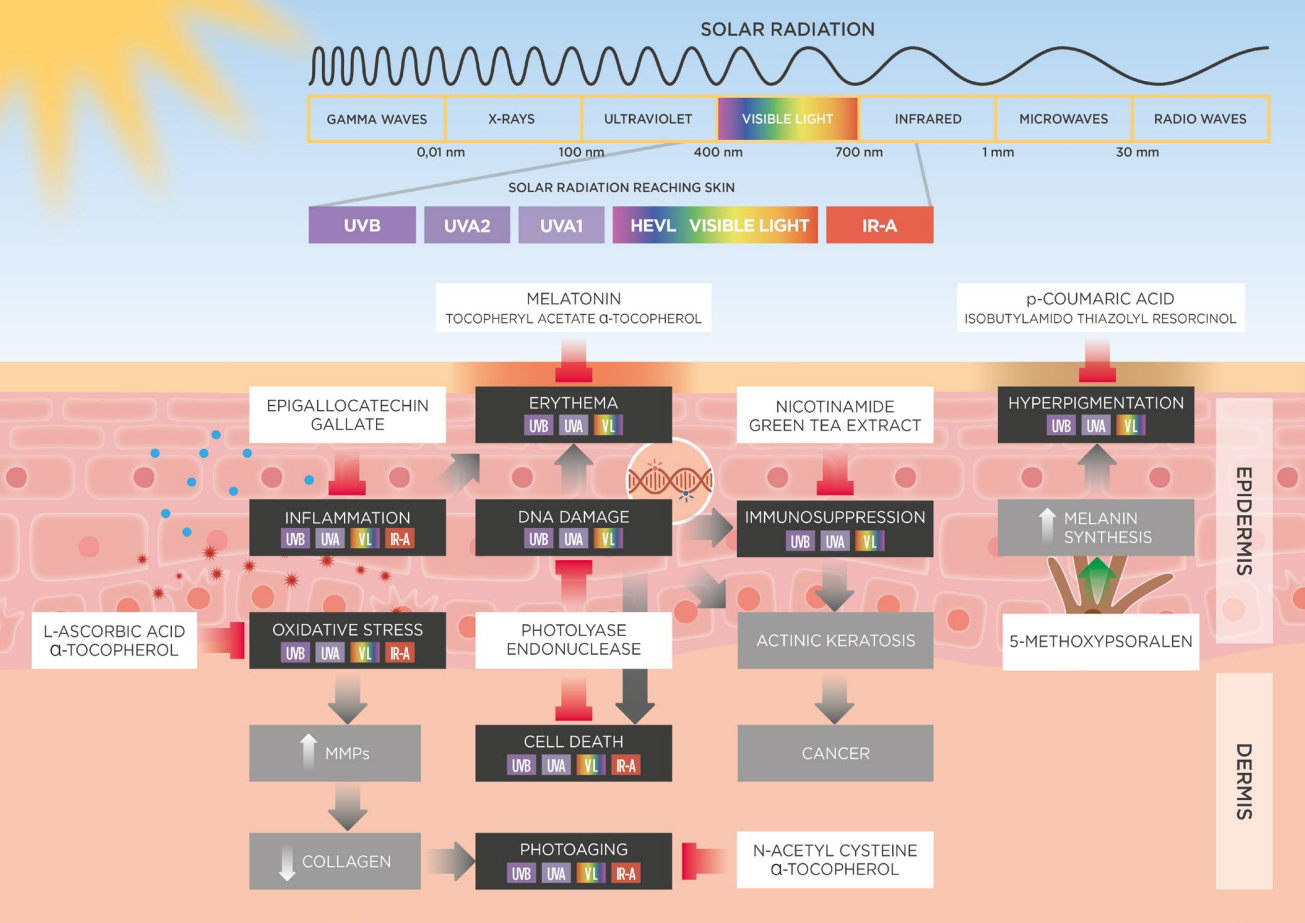
Top candidate PINGs and their molecular, cellular, and clinical targets.

Our findings indicated that, while PINGs generally enhance the performance of sunscreens, only a small subset is supported by high‐level evidence. The leading candidates are highlighted in Table 1 and Figure 1, with a summary of supporting clinical studies provided in Table 2. In the present review, we build upon that foundation by offering an in‐depth exploration of these top‐performing PINGs, which should be prioritized for inclusion in next‐generation sunscreen formulations. We detail their key properties, mechanisms of action, and roles in preventing or repairing sunlight‐induced skin damage. Additionally, we identify existing knowledge gaps and propose directions for future research.

**TABLE 1:** Mechanisms and evidence strength for top non‐filtering topical photoprotective ingredients.

Target	Optimal mechanism of action	Total number of photoprotective ingredients	Top ranked photoprotective ingredient(s)	Weight of evidence
Oxidative damage	Antioxidant	882	L‐Ascorbic acid	Very strong
			α‐Tocopherol	Very strong
DNA damage	DNA repair	353	Photolyase	Strong
			T4 DNA Endonuclease	Moderate
Immunosuppression	DNA repair	57	Nicotinamide	Very strong
			Green tea extract	Strong
Cell death	DNA repair	843	Photolyase	Moderate
Inflammation	Anti‐inflammatory	651	Epigallocatechin gallate	Strong
Erythema	Antioxidant	256	Tocopheryl acetate	Very strong
			Melatonin	Very strong
			α‐Tocopherol	Very strong
Pigmentation	Depigmenting	20	Isobutylamido thiazolyl resorcinol	Strong
			p‐Coumaric acid	Strong
Photoaging	Antioxidant	948	N‐Acetyl‐L‐cysteine	Very strong
			α‐Tocopherol	Moderate

*Note:* Evidence strength is based on available clinical, in vivo, and in vitro data as interpreted from Brown et al. [7] and supporting literature.

**TABLE 2:** Clinical studies supporting the top candidate PINGs.

Biological/clinical endpoint	Ingredient	Study	Subjects	Dose	Application schedule/site	Stress	Study type	Outcome
Oxidative stress	L‐ascorbic acid	Ou‐Yang et al. [8]	*N* = 7 Sex: Not specified Ethnicity: Not specified FST: II–IV Age: Not specified	0.1% and 3%	Pre: Applied 4 h before exposure Site: volar forearm	UVA 2 J/cm^2^	Placebo‐controlled	Treatment with 3% LAA significantly reduced UVA‐induced chemiluminescence
		Raschke et al. [9]	*N* = 23 Sex: Female (*n* = 23) Ethnicity: Not specified FST: Not specified Age (mean): 48.9 years	3%	Pre: Applied twice daily for 1 month Site: volar forearm	UVA 40 mJ/cm^2^	Placebo‐controlled	Significantly reduced UVA‐induced UPE 3% LAA was significantly more effective than 3% sodium ascorbyl phosphate
		Hagens et al. [10]	*N* = 20 Sex: Female (*n* = 20) Ethnicity: Not specified FST: Not specified Age: 18‐65 years	0.25% 0.5% 1.0%	Pre: Applied twice daily for 3 consecutive days Site: inner forearm	UVA 86 mJ/cm^2^	Placebo‐controlled	Significantly reduced UVA‐induced UPE at 0.5% and 1%
	α‐Tocopherol	Ley et al. [11]	*N* = 12 Sex: Not specified Ethnicity: Not specified FST: Not specified Age: Not specified	Not specified	Pre: Applied twice daily for 2 days Site: Back	UVA 10 J/cm^2^	Uncontrolled (no placebo)	Significantly inhibits squalene peroxidation by 28%
		Cho et al. [12]	*N* = 10 Sex: Female (*n* = 10) Ethnicity: Not specified FST: Not specified Age (range): 39–59 years Age (mean): 49.6 ± 7.6 years	5%	Pre: Applied 15 min before exposure Site: Forearm	UVA 30 J/cm^2^	Uncontrolled (no placebo)	Significantly reduced the level of carbonylated proteins in the stratum corneum by 41.45%
	
		Ekanayake‐Mudiyanselage et al. [13]	*N* = 13 Sex: Male (*n* = 5); Female (*n* = 13) Ethnicity: Not specified FST: I–IV Age (range): 21–33 years Age (mean): 27.7 years	0.15%	Pre: Subjects washed their skin with the formulation 30 min before exposure Site: Forearm	UVA 8 J/cm^2^	Placebo‐controlled	Significantly reduced squalene oxidation by 42% versus placebo
		Declercq et al. [14]	*N* = not specified Ethnicity: Not specified FST: II/III Age (range): 18–80 years	0.05%	1. Pre: Applied 1 h before exposure Site: Ventral forearm 2. Post: Applied immediately after exposure Site: Ventral forearm	UVA 1 J/cm^2^	Placebo‐controlled	1. Significantly reduced squalene oxidation by up to 82% versus vehicle when applied prior to UVR 2. No effect when applied after exposure
Ribet et al. [15]	*N* = 20 Sex: Male (*n* = 3); Female (*n* = 17) Ethnicity: Caucasian FST: II–IV Age (range): 25–54 years Age (mean): 39.1 ± 10.1 years	2%	Pre: Applied for 15 and 30 days before exposure Site: Leg (Thigh)	UVA 5 J/cm^2^	RCT (double‐blind)	13.6% and 12.1% reduction in levels of lipid peroxidation following 15 and 30 days of treatment, respectively
DNA damage	Photolyase	Stege et al. [16]	*N* = 19 Sex: Not specified Ethnicity: Not specified FST: II/III Age: Adult	1% Photolyase‐containing liposomes	Post: Applied immediately after irradiation for 60 min before photoreactivation Site: Buttock	UVB 1 × MED Photoreaction: 340‐450 nm for 30 min at 9.6 mW/cm^2^	Uncontrolled (no placebo)	Significant reduction in number of CPDs in the epidermis after photoreactivation Efficacy dependent upon time of exposure to photoreactivating light with maximal efficacy (40%–45% reduction in CPD count) at 30 min
		Berardesca et al. [17]	*N* = 10 Sex: Male (*n* = 5); Female (*n* = 5) Ethnicity: Caucasian FST: II Age (range): 36–79 years Age (mean): 63.5 years	SPF50 sunscreen containing 1% liposome‐encapsulated photolyase	Pre: Applied 30 before irradiation Site: Lower back	SSR 3 × MED for 4 consecutive days	Placebo‐controlled	Photolyase‐containing sunscreen reduced number of CPDs by 93%. Sunscreen alone reduced CPDs by only 62%.
	T4 DNA Endonuclease	Wolf et al. 2000 [18]	*N* = 15 Sex: Male (*n* = 13); Female (*n* = 2) Ethnicity: FPT: Age (range): years	Not specified	Post: Applied immediately after irradiation Site: Buttock	SSR 2 × MED	Placebo‐controlled	T4N5 liposomes prevented UVR‐induced upregulation of IL‐10 and TNF‐α and demonstrated a trend towards repair of DNA damage.
Immunosuppression (Elicitation phase)	Nicotinamide	Sivapirabu et al. [19]	*N* = 17 Sex: Male (*n* = 2); Female (*n* = 15) Ethnicity: Not specified Age (range): 24–66 years Age (median): 37 years	5%	Post: Applied immediately after irradiation Site: Back	Single exposure to 1, 2 and 4 J/cm^2^ SSR	RCT (double‐blind)	Significantly reduced immunosuppression at all doses as evaluated by measuring Mantoux‐induced erythema.
	
		Sivapirabu et al. [19]	*N* = 18 Sex: Male (*n* = 5); Female (*n* = 17) Ethnicity: Not specified Age (range): 22–70 years Age (median): 39 years	5%	Post: Applied immediately after irradiation Site: Back	SSR once daily for 3 consecutive days 1, 2 and 4 J/cm^2^	RCT (double‐blind)	Significantly reduced immunosuppression at all doses as evaluated by measuring Mantoux‐induced erythema
		Sivapirabu et al. [19]	*N* = 20 Sex: Male (*n* = 8); Female (*n* = 12) Ethnicity: Not specified Age (range): 23–54 years Age (median): 37 years	0.2%	Post: Applied immediately after irradiation Site: Back	SSR once daily for 3 consecutive days 1, 2 and 4 J/cm^2^	RCT (double‐blind)	Significantly reduced immunosuppression at all doses by 28%, 46%, and 62%, respectively as evaluated by measuring Mantoux‐induced erythema
		Sivapirabu et al. [19]	*N* = 15 Sex: Male (*n* = 3); Female (*n* = 12) Ethnicity: Not specified Age (range): 23–51 years Age (median): 34 years	5%	Post: Applied immediately after irradiation Site: Back	UVA 300 and 600 mJ/cm^2^	RCT (double‐blind)	Significantly reduced immunosuppression at low dose but not at high dose as evaluated by measuring Mantoux‐induced erythema
Sivapirabu et al. [19]	*N* = 15 Sex: Male (*n* = 3); Female (*n* = 12) Ethnicity: Not specified Age (range): 23–51 years Age (median): 34 years	5%	Post: Applied immediately after irradiation Site: Back	UVB 60 mJ/cm^2^	RCT (double‐blind)	Significantly reduced immunosuppression as evaluated by measuring Mantoux‐induced erythema
	
		Damian et al. [20]	*N* = 20 Sex: Male (*n* = 10); Female (*n* = 10) Ethnicity: Not specified FST: I–III Age (range): 22–63 years Age (median): 35 years	5%	1. Pre: Applied 15 min before irradiation Site: Upper back 2. Post: Applied immediately after irradiation Site: Upper back	s 0.74, 1.48 or 2.22 J/cm^2^ for 3 consecutive days	RCT (double‐blind)	1. Significantly reduced immunosuppression as evaluated by measuring Mantoux‐induced erythema 2. Significantly reduced immunosuppression as evaluated by measuring Mantoux‐induced erythema
		Damian et al. [20]	*N* = 18 Sex: Female (*n* = 18) Ethnicity: Not specified FST: I–III Age (range): 25–63 years Age (median): 41 years	5%	Pre/Post: Applied 15 min before and immediately after irradiation Site: Upper back	SSR 0.74, 1.48 or 2.22 J/cm^2^ for 3 consecutive days	RCT (double‐blind)	Immunosuppression induced only at the highest SSR dose NAM significantly reduced immunosuppression as evaluated by measuring Mantoux‐induced erythema
Damian et al. [20]	*N* = 18 Sex: Male (*n* = 18) Ethnicity: Not specified FST: I–III Age (range): 23–56 years Age (median): 32 years	5%	Pre/Post: Applied 15 min before and immediately after irradiation Site: Upper back	SSR 0.74, 1.48 or 2.22 J/cm^2^ for 3 consecutive days	RCT (double‐blind)	Immunosuppression induced at all 3 doses. NAM significantly reduced immunosuppression.
	Green tea extract	Li et al. 2009 [21]	*N* = 20 Sex: Female (*n* = 20) Ethnicity: Chinese FPT: III/IV Age: Not specified	2% 3% 4% 5%	Pre/Post: Applied 30 min before and 6, 24, and 48 h after irradiation Site: Back	SSR 1.5 × MED	Placebo‐controlled	3% significantly prevented loss of CD1a^+^ cells from skin following UVR exposure All other concentrations had no effect
		Camouse et al. 2009 ^60^	*N* = 10 Sex: Not specified Ethnicity: Not specified FPT: I–III Age: Not specified	2.5 mg/cm^2^	Pre/Post: Applied 15 min before and immediately after irradiation Site: Buttock	SSR 2 × MED	RCT	Significantly prevented loss of CD1a^+^ cells from skin following UVR exposure
Cell death (Apoptosis)	Photolyase	Stege et al. [16]	*N* = 19 Sex: Not specified Ethnicity: Not specified FST: II/III Age: Adult	1% Photolyase‐containing liposomes	Post: Applied immediately after irradiation for 60 min before photoreactivation Site: Buttock	UVB 1 × MED Photoreaction: 340‐450 nm for 30 min at 9.6 mW/cm^2^	Uncontrolled (no placebo)	Completely prevented sunburn cell formation
		Berardesca et al. [17]	*N* = 10 Sex: Male (*n* = 5); Female (*n* = 5) Ethnicity: Caucasian FST: II Age (range): 36–79 years Age (mean): 63.5 years	SPF50 sunscreen containing 1% liposome‐encapsulated photolyase	Pre: Applied 30 before irradiation Site: Lower back	SSR 3 × MED for 4 consecutive days	Placebo‐controlled	SS alone significantly, but not completely, prevented CPD formation, reducing it by 40% (*p* < 0.001). Topical SS + photolyase prevented apoptosis by ~82% (*p* < 0.001).
Inflammation	Epigallocatechin gallate	Katiyar et al. [22]	*N* = 6 Sex: Male/Female Ethnicity: Caucasian FST: II/III Age: 25–56 years	1 m/cm^2^	Pre: Applied 20 min before exposure Site: Buttock	UVB 4 × MED	Uncontrolled (no placebo)	Significantly reduced the infiltration of CD11b^+^ cells
Erythema	Tocopheryl acetate	Zhai et al. [23]	*N* = 10 Sex: Male (*n* = 3); Female (*n* = 7) Ethnicity: Caucasian FST: II/III Age (mean): 47 ± 10 years	2.3%	Pre: Applied 30 min before UVR exposure Site: Forearm	UVB (54%) UVA (43%) UVC (3%) 1 × MED	RCT (double‐blind)	Significantly reduced erythema on Days 2 and 3 after UVR exposure

	α‐Tocopherol	Perugini et al. [24]	*N* = 10 Sex: Female (*n* = 10) Ethnicity: Not specified FPT: II/III Age (range): 20–30 years	Not specified	Pre: Applied 30 min before UVR exposure Site: Buttock	UVB 2 × MED	Uncontrolled (No placebo)	Reduced erythema by 57.7%
		Dreher et al. [25]	*N* = 12 Sex: Male (*n* = 6); Female (*n* = 6) Ethnicity: Caucasian FPT: II/III Age (range): 29–49 years Age (mean): 40 ± 7 years	2%	Pre: Applied 30 min before UVR exposure Site: Lower back	UVB (54%) UVA (43%) UVC (3%) 1 × MED	RCT (double‐blind)	Significantly reduced erythema after 24 h as assessed by chronometer and by measuring blood flow
	Melatonin	Dreher et al. [25]	*N* = 12 Sex: Male (*n* = 6); Female (*n* = 6) Ethnicity: Caucasian FPT: II/III Age (range): 29–49 years Age (mean): 40 ± 7 years	1% 2.5%	Pre: Applied 30 min before UVR exposure Site: Lower back	UVB 1 × MED	Placebo‐controlled	Dose‐dependent reduction in the severity of erythema
		Bangha et al. [26]	*N* = 20 Sex: Male (*n* = 5); Female (*n* = 15) Ethnicity: Not specified FPT: II/III Age (range): 22–33 years Age (median) 27 years	0.5%	1. Pre: Applied 15 min before UVR exposure Site: Lower back 2. Post: Applied 1, 30, or 240 min after exposure Site: Lower back	UVB/A 2 × MED	RCT (double‐blind)	1. Significant suppression of erythema when applied prior to exposure 2. No effect when applied after exposure
Fischer et al. [27]		*N* = 20 Sex: Male (*n* = 5); Female (*n* = 15) Ethnicity: Not specified FPT: II/III Age (range): 22–33 years Age (median): 27 years	0.5%	1. Pre: Applied 15 min before UVR exposure Site: Lower back 2. Post: Applied 1, 30, or 240 min after exposure Site: Lower back	UVB/A Dose not specified	RCT (double‐blind)	1. Significant suppression of erythema when applied prior to exposure 2. No effect when applied after exposure
	
		Scheuer et al. [28]	*N* = 23 Sex: Male (*n* = 7); Female (*n* = 15) Ethnicity: Not specified FPT: I–III Age (range): 24–53 years Age (median): 32 years	0.5% 2.5% 12.5%	Pre Site: Back	Natural sunlight for 40 min	RCT (double‐blind)	Significant suppression of erythema compared to placebo for 12.5% cream 0.5% and 2.5% were ineffective
Scheuer et al. [29]	*N* = 23 Sex: Not specified Ethnicity: Not specified FPT: Not specified Age: Not specified	0.5% 2.5% 12.5%	Pre Site: Back	Natural sunlight for 40 min (UV index 9)	RCT (double‐blind)	Significant suppression of erythema compared to placebo for 12.5% cream 0.5% and 2.5% were ineffective
Pigmentation	Isobutylamido thiazolyl resorcinol	Vachiramon et al. [30]	*N* = 30 Sex: Male (*n* = 1); Female (*n* = 29) FST: II–IV Age: 34.77 ± 9.6 years	0.15%	Pre: Applied for 3 weeks prior to exposure to UVB Site: Inner upper arm	210, 270, and 300 mJ/cm^2^ UVB	RCT (single‐blinded)	ITR‐treated sides showed a statistically significant lower mean lightness index compared to control and returned to normal skin color earlier than the control side (*P* < 0.05).
	p‐Coumaric acid	Seo et al. [31]	*N* = 21 Sex: Not specified Ethnicity: Not specified FST: III–IV Age (mean): 35.2 ± 6.79 years Age (range): 18–60 years	1.5%	Pre/Post	Up to 5 × MED	RCT (double‐blind)	Melanin index significantly decreased in PCA‐treated skin exposed to UVR compared to control cream. Increase in ITA value was also significantly greater.
Photoaging	N‐acetyl‐L‐cysteine	Kang et al. [32]	*N* = Not specified Sex: Not specified Ethnicity: Not specified FST: Not specified Age: Not specified	20%	Pre: Applied under occlusion 24 h prior to UVR exposure Site: Buttock	SSR 2 × MED	Placebo‐controlled	Inhibits MMP‐1 expression. Inhibitions MAPK signaling.
		Jin et al. [33]	*N* = 13 Sex: Male (*n* = 5); Female (*n* = 8) Ethnicity: Caucasian FST: Not specified Age (range): 20–29 years	20%	Pre: Applied under occlusion 24 h prior to UVR exposure Site: Buttock	UVB + UVA 2 × MED	Placebo‐controlled	Significantly reduces MMP‐12 expression (−54%).
		Qin et al. [34]	*N* = Not specified Sex: Not specified Ethnicity: Not specified FST: Not specified Age: Not specified	20%	Pre: Applied under occlusion 24 h prior to UVR exposure Site: Buttock	SSR 2 × MED	Placebo‐controlled	Significantly reduces c‐Jun expression by 60%. Significantly reduces CCN1 expression by 65%. Significantly prevents loss of type I collagen expression by 50%.

Abbreviations: Bax, Apoptosis regulator BAX; Bcl‐2, B‐cell lymphoma 2; CCN1, Cellular Communication Network Factor 1; c‐Jun, Transcription factor Jun; CPD, Cyclobutane pyrimidine dimer; FPT, Fitzpatrick skin phototype; LAA, L‐ascorbic acid; MAPK, Mitogen activated protein kinase; MED, Minimal Erythema Dose; MMP, Matrix metalloproteinase; NAM, Nicotinamide; RCT, Randomized controlled trial; SSR, solar‐simulated radiation; UPE, Ultraweak photon emission.

## 2 Methods

This review builds upon our previously published evidence‐based classification of over 1700 topical photoprotective ingredients (PINGs), in which we ranked compounds according to the type and volume of experimental support for their photoprotective capacity [7]. This methodology ranked evidence according to experimental rigor, translatability, and internal validity, with a weighting scale from in vitro studies (score = 1) to randomized controlled trials (score = 20). These scores were aggregated and capped based on the highest level of support to prevent overemphasis on low‐validity studies. The final scores were then used to assign a “Weight of Evidence” classification ranging from “Very Weak” to “Very Strong.”

For the current analysis, we conducted a targeted literature review to reassess the top‐ranked PINGs, focusing on those with the strongest evidence from human studies. Ingredients were included if they demonstrated biological activity relevant to photodamage prevention or repair, such as antioxidant, DNA repair, immunomodulatory, anti‐inflammatory, or pigmentation‐regulating effects. We prioritized substances compatible with topical use and suitable for incorporation into sunscreen formulations.

Each selected PING was evaluated across seven clinical and biological endpoints of solar skin damage: oxidative stress, DNA damage, immunosuppression, apoptosis, inflammation, erythema, and pigmentation. For each endpoint, the strength of evidence was reassessed by reviewing available peer‐reviewed studies, with emphasis on randomized controlled trials, mechanistic studies, and controlled human or ex vivo models.

Our analysis aimed to (i) identify ingredients with the highest translational potential, (ii) highlight formulation and mechanistic considerations for their use in sunscreens, and (iii) identify research gaps, especially in areas of VL and IR protection, where standard evaluation frameworks remain limited.

## 3 Results

### 3.1 The Impact of Sunlight on Human Skin

Human skin is in continuous contact with the external environment and is thus uniquely vulnerable to solar radiation. Although sunlight is essential for circadian regulation and cutaneous vitamin D synthesis, chronic and unprotected exposure poses significant risks to skin health. The effects of sunlight on skin, however, are complex and multifaceted, and each wavelength affects the skin differently depending on its energy, penetration depth, abundance, and interaction with chromophores. These interactions trigger molecular and cellular changes that lead to both immediate effects (e.g., erythema) and long‐term damage (e.g., photoaging and cancer).

While most sunscreen filters only protect against UVR, the effects of sunlight exposure are not confined to the UV spectrum but are now known to extend across VL and IR, with cumulative interactions leading to oxidative stress, DNA damage, immune suppression, extracellular matrix (ECM) degradation, pigmentation disturbances, and carcinogenesis. Other exposome factors, including synergistic damage from pollutants, also further compromise skin integrity.

UVR is the most studied component of solar radiation. It ranges from approximately 100 to 400 nm (nm) and is traditionally divided into UVC (100–280 nm), UVB (280–320 nm), and UVA (320–400 nm). UVC radiation represents the most energetic and potentially damaging portion of the solar UV spectrum, capable of inducing severe DNA damage and cellular destruction. However, under natural conditions, UVC is entirely absorbed by atmospheric ozone and oxygen, and thus does not reach the Earth's surface. UVB radiation, in contrast, accounts for approximately 5% of UVR reaching the skin. It is primarily absorbed by the epidermis, where it exerts direct photochemical damage to cellular DNA. Absorption of UVB photons damages DNA both directly, by forming thymine dimers (TDs) such as cyclobutane pyrimidine dimers (CPDs) and pyrimidine‐pyrimidine (6‐4) photoproducts (6‐4PPs), and indirectly through oxidative stress, leading to lesions like 8‐hydroxydeoxyguanosine (8‐OHdG) and DNA strand breaks [35, 36, 37, 38]. These mutagenic lesions disrupt base pairing and, if not efficiently repaired, result in mutations in tumor suppressor genes such as p53 that can lead to the development of actinic keratoses (AK), squamous cell carcinoma, and basal cell carcinoma. To eliminate such damage and maintain genomic stability, skin is equipped with a comprehensive genomic surveillance and DNA repair system; primarily base excision repair (BER) for small oxidative lesions and nucleotide excision repair (NER) for bulky dimers [39, 40, 41].

UVB exposure also triggers an acute inflammatory response characterized by keratinocyte apoptosis (sunburn cells [SBCs]), cytokine release (e.g., IL‐1, TNF‐α), and vasodilation, clinically manifesting as sunburn [27, 42, 43]. Prolonged or repeated exposure also promotes skin thickening (epidermal hyperplasia) as a defense mechanism but simultaneously accelerates cumulative photodamage.

UVA radiation, accounting for over 95% of UV radiation reaching the skin, penetrates more deeply into the dermis. It is subdivided into UVA2 (320–340 nm) and UVA1 (340–400 nm). Unlike UVB, UVA causes indirect DNA damage primarily through the generation of reactive oxygen species (ROS), such as superoxide anions, hydroxyl radicals, and hydrogen peroxide. These ROS interact with cellular lipids, proteins, and nucleic acids, initiating oxidative modifications that compromise cellular integrity and promote mutagenesis. While CPDs are traditionally attributed to UVB‐induced direct DNA damage, growing evidence indicates that UVA can also induce CPDs, which may continue to form for up to 24 h after exposure [44]. These delayed lesions, known as dark‐CPDs, account for over half of all CPDs formed in melanocytes [45]. They result from chemiexcitation of melanin triggered by peroxynitrite (ONOO‐), a reactive byproduct of ROS [45]. UVA1 also activates signal transduction pathways such as MAPKs and AP‐1, leading to the upregulation of matrix metalloproteinases (MMPs), particularly MMP‐1 and MMP‐9. These enzymes degrade type I and III collagen and other structural ECM proteins. UVA1 exposure also inhibits new collagen synthesis [46], and together these effects reduce skin elasticity and strength, leading to wrinkling and sagging.

UVR‐induced CPD formation is also a key trigger for immune suppression [47]. DNA damage prompts Langerhans cells (LCs) to migrate to lymph nodes, where they activate regulatory T cells to release immunosuppressive cytokines like interleukin (IL)‐10 and tumor necrosis factor‐α (TNF‐α) [48], impairing antigen presentation and skewing cytokine profiles toward immunoregulatory pathways. This immunomodulation increases susceptibility to infections, tumor progression, and delayed hypersensitivity responses.

Moreover, both UVB and UVA stimulate melanogenesis via paracrine signaling and oxidative stress, resulting in persistent pigment darkening and the development of lentigines, particularly in sun‐exposed areas. Melanin production in response to sunlight is an adaptive mechanism that protects the skin from further damage. In humans, pigmentation occurs via two distinct mechanisms: immediate tanning, which is short‐lived and not photoprotective [49, 50], and delayed tanning, which involves melanin synthesis and provides lasting protection [51]. Two chemically and functionally distinct types of melanin are synthesized within melanocytes. Black to brown eumelanin is a redox and UV‐absorbing agent that can scavenge ROS and dissipate over 99.9% of absorbed UVR and VIS. In contrast, yellow to reddish‐brown pheomelanin is highly photo‐reactive, enhancing the UVR‐induced production of ROS within keratinocytes [52].

Recent research has also highlighted the detrimental effects of VL (400–700 nm), especially high‐energy visible blue light (HEVL; 400–500 nm), on skin physiology. Although traditionally regarded as biologically inert compared to UV radiation, HEVL has been shown to induce melanogenesis and to trigger ROS production in mitochondria [53]. Exposure to high‐energy visible light (HEVL), particularly in darker phototypes (Fitzpatrick skin type [FST] IV–VI), triggers long‐lasting pigmentation [54, 55]. In vitro and ex vivo studies have demonstrated that melanin synthesis induced by HEVL is mediated through the activation of Opsin 3 (OPN3), a photoreceptor expressed on melanocytes, resulting in the formation of a multimeric tyrosinase/tyrosinase‐related protein complex and sustained tyrosinase activity [56].

IR‐A radiation (700–1400 nm), the most abundant portion of solar radiation in terms of energy, penetrates deeply into the dermis and subcutaneous layers. Unlike UV and VL, IR‐A interacts with relatively few discrete chromophores, most notably mitochondrial cytochrome c oxidase, but these interactions are biologically significant because they enhance mitochondrial ROS generation and activate downstream pathways that contribute to extracellular matrix degradation and photoaging [57, 58, 59].

In contrast to UVB, VL‐ and IR‐A–induced effects generally require radiant exposures that are 10^3^–10^5^‐fold higher than those of UVB to elicit clinically relevant outcomes, such as persistent pigmentation (VL: ~40–480 J/cm^2^) or MMP‐1 upregulation (IR‐A: ~360–720 J/cm^2^) [54, 55, 57, 60]. While VL and IR‐A are therefore less efficient on a per‐photon basis, their abundance and cumulative exposures make them important contributors to photoaging and pigmentation, supporting the rationale for developing PINGs that target these wavebands. Emphasizing this, VL and IR together have been estimated to account for roughly one‐half of sunlight‐induced ROS, with the remainder arising mainly from UVA [4].

Clinically, chronic exposure to solar radiation results in a spectrum of disorders. Photoaging is characterized by fine and coarse wrinkling, rough texture, loss of elasticity, telangiectasias, and irregular pigmentation. These signs reflect underlying changes in dermal collagen organization, glycosaminoglycan depletion, and persistent inflammatory infiltration. Pigmentary changes, including solar lentigines, melasma, and ephelides, are prevalent in both fair‐ and dark‐skinned individuals, with the latter showing more persistent and pronounced hyperpigmentation. Photocarcinogenesis remains one of the most serious consequences of solar exposure. UVB is the primary initiator of keratinocyte cancers through its direct mutagenic effects, while UVA contributes to melanoma risk via oxidative DNA damage and immune evasion mechanisms.

The biological and clinical consequences of solar exposure can be specifically addressed using targeted PINGs, alone or in combination; however, no single PING provides comprehensive protection.

### 3.2 PINGs With Antioxidative Properties

All wavelengths of solar radiation, especially UVA1 and IR‐A, generate ROS through interactions with skin chromophores. These excited chromophores subsequently transfer energy or electrons to oxygen, forming ROS, which contribute to oxidative stress and skin damage [61]. The skin defends against ROS using water‐ and lipid‐soluble antioxidants (e.g., vitamins A, C, and E) and enzymatic systems. However, levels of these antioxidant defenses decline with age and UVR exposure, reducing the skin's ability to neutralize ROS and reactive nitrogen species (RNS) [62, 63], leading to a state of redox imbalance known as oxidative distress. Topical application of antioxidants or agents that boost endogenous antioxidant enzyme activity is therefore an attractive photoprotective strategy.

Antioxidants comprised over half of all identified PINGs in the study of Brown et al. [7], but only 15 (1.7%) of these had clinical support (Table 3). Nevertheless, the evidence for adding antioxidants to sunscreens is compelling: sunscreens supplemented with antioxidants have shown superior protection against UVR‐ and IR‐A‐induced MMP expression compared to sunscreens containing filters alone [74, 75, 76].

**TABLE 3:** Clinically validated antioxidant substances.

Ingredient	Efficacious dose	Stress	References
3,4‐Dihydro‐3‐(4‐hydroxyphenyl)‐2H‐1‐benzopyran‐7‐ol (NV‐07α)	0.005%	UVB/A	Widyarini et al. [64]
α‐Glycosylrutin	0.25%	UVA	Hagens et al. [10]
α‐Tocopherol	0.15%	UVA	Ekanayake‐Mudiyanselage et al. [13]
	0.05%	UVA	Declercq et al. [14]
	0.2%	UVA	Ley [11]
	2%	UVA	Ribet et al. [15]
	5%	UVA	Cho et al. [12]
Argania spinosa extract	3%	UVB/A	Danoux et al. [65]
β‐Carotene	0.2%	IRA	Darvin et al. [66]
	5%	UVA	Cho et al. [12]
Carboxymethylated beta‐(1‐3)‐glucan	2%	UVA	Zϋlli et al. [67]
Epigallocatechin gallate	Not specified	UVB	Katiyar et al. [22]
EUK‐134	0.05% and 0.1%	UVA	Declercq et al. [14]
Grape seed proanthocyanidins	Not specified	UVA	Van Wijk et al. [68]
*Hypericum perforatum* extract	1.5%	VIS/IRA	Arndt et al. [69]
	1.5%	VIS/IRA	Haag et al. [70]
L‐Ascorbic acid	3%	UVA	Ou‐Yang et al. [8]
	3%	UVA	Raschke et al. [9]
	0.5% and 1%	UVA	Hagens et al. [10]
Licorice extract	Not specified	UVA	Kühnl et al. [71]
N‐(3,4‐Dihydroxybenzyl)‐2‐(4‐hydroxy‐3‐methoxyphenyl)acetic acid	0.1%	UVA	Mann et al. [72]
Sodium ascorbyl phosphate	3%	UVA	Raschke et al. [9]
Tocopheryl acetate	Not specified	UVA	Belli et al. [73]

*Note:* Evidence strength is based on available clinical, in vivo, and in vitro data from Brown et al. [7].

The top ranked PINGs, L‐ascorbic acid (LAA; vitamin C) and α‐Tocopherol (TOC; vitamin E) (Table 1), both form part of skin's endogenous antioxidant network and work cooperatively to detoxify the skin: oxidized vitamin E is regenerated by vitamin C, which itself is regenerated by glutathione, maintaining an active antioxidant reservoir in the skin [77]. Indeed, combining these two substances has been shown to be even more effective than using either substance alone. The combination of 15% LAA with 1% TOC, for example, quadruples its photoprotective capacity compared to LAA alone [77]. LAA, however, is inherently unstable and exhibits poor penetration through the lipophilic stratum corneum. Derivatives like sodium ascorbyl phosphate or α‐tocopherol phosphate are more stable but less effective because they first must undergo enzymatic hydrolyzation in the skin to release pure ascorbic acid [9, 78]. For better delivery and stability, nanocarriers like spanlastics have been used successfully: encapsulated LAA showed enhanced skin penetration, remained stable over 6 months, and significantly reduced MMP‐2 and MMP‐9 expression in UVB‐exposed rats compared to unencapsulated formulations [79].

### 3.3 PINGs That Support DNA Repair

While the NER pathway is generally effective at correcting UVR‐induced TDs, its efficiency varies significantly between lesion types. Specifically, repair of CPDs is notably slower and less efficient than that of 6‐4PPs [80, 81], leading to their persistence in DNA and potential propagation of mutations during replication. Compounding this vulnerability, the capacity of DNA repair mechanisms declines progressively with age, estimated at approximately 1% per year [82, 83]. To address these deficits, topical application of DNA repair enzymes like photolyase (EC 4.1.99.3) and endonuclease (EC 3.1.25.1) has emerged as a promising photoprotection strategy.

Photolyase repairs CPDs by directly cleaving TDs in a VL‐dependent process known as photoreactivation. Stege et al. [16] demonstrated that topical application of 1% liposome‐encapsulated recombinant photolyase from *Anacystis nidulans* reduced CPD levels by 40%–45% following UVB exposure and subsequent photoreactivation, while also preventing UVB‐induced erythema and immunosuppression. In a separate study, Berardesca et al. [17] showed that an SPF50 sunscreen containing photolyase significantly outperformed the same sunscreen without it, increasing CPD protection from 62% to 93% after exposure to solar‐simulated radiation (SSR). Additional evidence suggests that SPF100+ formulations with photolyase are more effective than UV filters alone in reducing AK [84].

Topical application of liposomal bacteriophage T4 endonuclease 5 (T4N5; Dimericine) was also shown to enhance dimer repair in human skin in vivo [18], and reduce both the incidence and yield of skin cancer in mice [85, 86]. In clinical trials with patients with xeroderma pigmentosum, a rare autosomal recessive disease characterized by defective NER, topical application of T4N5 reduced the incidence of basal cell carcinoma by 30% and of AK by more than 68% [87]. Similarly, *Micrococcus luteus* endonuclease prevents telomere shortening and c‐fos oncogene overexpression: effects fully blocked when combined with photolyase‐containing sunscreen [88].

Antioxidants have also emerged as critical tools in preventing the formation of dark‐CPDs. Pretreatment with *Polypodium leucotomos* extract (PLE), a polyphenol‐rich botanical derived from tropical fern leaves, or with TOC, has been shown to significantly inhibit dark‐CPD formation in both murine melanocytes and human keratinocytes following UVA irradiation [45, 89, 90]. As such, antioxidant‐enriched sunscreens offer an important complementary strategy, particularly for mitigating persistent photodamage in pigmented skin.

### 3.4 PINGs That Counteract Immunosuppression

Enhancing DNA repair can not only reduce mutagenesis but can also mitigate the immunosuppressive effects of UVR. For example, topical application of liposome‐encapsulated photolyase has been shown to restore contact hypersensitivity responses to nickel sulfate in UVB‐irradiated human skin [16], indicating functional recovery of cutaneous immune surveillance during the elicitation phase of the response. UV‐induced immunosuppression during the sensitization phase is also a central mechanism linking solar exposure to skin cancer risk [48].

Two promising sunscreen‐compatible PINGs, nicotinamide (NAM) and green tea extract (GTE) (Table 1), also exert key photoprotective effects by enhancing DNA repair pathways. NAM, a vitamin B3 derivative and precursor of nicotinamide adenine dinucleotide (NAD^+^), supports ATP‐dependent repair processes and helps maintain cellular energy balance, which is critical for effective nucleotide excision repair. NAM has been shown to enhance DNA repair and reduce the formation of both CPDs and 8‐OHdG in UV‐irradiated keratinocytes [91]. These protective effects are primarily attributed to NAM's ability to elevate intracellular NAD^+^ levels, thereby activating poly(ADP‐ribose) polymerase 1 (PARP1), which facilitates chromatin remodeling and DNA repair complex recruitment [92]. Clinically, topical NAM at concentrations of 0.2% and 5% has been demonstrated to prevent SSR‐induced immunosuppression, as assessed by the preservation of Mantoux‐induced erythema responses [19, 20], again reflecting protection of the elicitation phase.

GTE contributes to photoprotection by modulating redox‐sensitive signaling pathways and upregulating DNA repair enzyme expression. Camouse et al. [93] and Li et al. [21] demonstrated that topical application of GTE partially inhibited the emigration of CD1a^+^ Langerhans cells from human epidermis following SSR exposure. Additionally, subjects treated with GTE showed enhanced contact hypersensitivity responses to dinitrochlorobenzene after exposure to 0.75× and 2× MED SSR, compared to unprotected controls [93], consistent with preserved challenge (elicitation) responses. GTE is rich in polyphenols (green tea polyphenols; GTPs) and notably, mice administered GTPs and its principal bioactive component epigallocatechin gallate (EGCG) displayed fewer CPD‐positive LCs in their draining lymph nodes following UVR exposure than their non‐GTP‐treated counterparts [94, 95]. GTPs have also been shown to enhance the expression of NER genes (XPA, XPC, RPA1) at UVB‐exposed skin sites and to accelerate CPD repair in cell culture, animal models, and human skin [95, 96].

### 3.5 PINGs That Reduce Apoptosis

Apoptosis of UV‐damaged keratinocytes is a natural protective mechanism that prevents the survival of cells carrying potentially mutagenic DNA lesions [97, 98]. By limiting oxidative stress and enhancing DNA repair, PINGs reduce DNA damage and thereby reduce apoptosis. In this context, the reduction of apoptosis—and consequently the formation of sunburn cells (SBCs), which serve as histological markers of severe photodamage—can be used as a marker of photoprotection. For example, Stege et al. [16] demonstrated that treatment with photolyase‐containing liposomes followed by photoreactivation completely prevented the formation of SBCs in UVB‐irradiated skin. Similarly, a photolyase‐enriched sunscreen provided significantly better protection against apoptosis than sunscreen alone, increasing efficacy from 40% to 82% [17].

In parallel, compatible solutes, such as ectoine, betaine, and mannitol, offer a complementary strategy by stabilizing cellular structures under environmental stress. These molecules form protective hydration shells around proteins, DNA, and lipids, thereby preserving their functional conformation [99]. Among them, ectoine has been shown to reduce SBC formation and inhibit UVR‐triggered LC migration, preserving epidermal immune function [100]. It also protects against mitochondrial DNA damage in UVA‐exposed skin [101]. Importantly, an SPF30 sunscreen supplemented with ectoine and mannitol provided significantly better protection than an equivalent formulation without these solutes [102].

A key concern is whether reducing apoptosis might allow survival of cells carrying DNA mutations and thereby increase cancer risk. However, available evidence suggests this is not the case for calcitriol (1,25‐dihydroxyvitamin D_3_; 1,25(OH)_2_D_3_), the active form of vitamin D_3_. Rather than suppressing apoptosis directly, calcitriol reduces UVR‐induced DNA damage by limiting oxidative stress and enhancing repair. Consistently, treatment has been associated with lower levels of UVR‐induced DNA lesions and reduced rates of skin tumor development in mice [103, 104], supporting the protective potential of PINGs.

### 3.6 PINGs With Anti‐Inflammatory Properties

Sunlight‐induced inflammation is primarily driven by oxidative stress and the activation of transcription factors such as NF‐κB and AP‐1, which regulate the expression of a broad array of pro‐inflammatory genes. Among the top‐ranked PINGs compatible with sunscreen formulations, EGCG is noted for its potent immunomodulatory effects [105]. EGCG has been shown to inhibit NF‐κB signaling, thereby dampening the transcriptional activation of inflammatory mediators. It also suppresses the expression of inducible nitric oxide synthase (iNOS) and cyclooxygenase‐2 (COX‐2), two key enzymes involved in the amplification of UVR‐induced inflammatory cascades [105, 106]. In parallel, EGCG exerts antioxidant activity, neutralizing ROS and RNS that serve as upstream triggers of inflammatory gene expression. Collectively, these actions lead to a marked reduction in epidermal hyperplasia, leukocyte infiltration, and cutaneous edema in both human and murine models of UVR‐induced skin inflammation [22, 107]. These findings support EGCG as a multifunctional PING capable of interrupting the molecular and cellular drivers of photo‐induced inflammation, making it an ideal adjunct to traditional sunscreen filters.

### 3.7 PINGs That Reduce Erythema

Given the multifactorial nature of erythema, a variety of pharmacological and bioactive agents have demonstrated efficacy in mitigating its development. These include both steroidal anti‐inflammatory drugs, such as hydrocortisone, betamethasone, and methylprednisolone, and non‐steroidal agents like indomethacin and diclofenac. With relevance to sunscreens, antioxidants, including TOC, tocopheryl acetate, and melatonin, have shown significant anti‐erythema effects by scavenging ROS and modulating redox‐sensitive signaling pathways [23, 24, 25, 108, 109]. Finally, DNA repair enzymes such as photolyase have proven effective by reducing underlying UVR‐induced DNA damage that contributes to inflammatory signaling [16]. These diverse mechanisms highlight the therapeutic potential of combining anti‐inflammatory, antioxidant, and DNA‐repair strategies for optimal control of UVR‐induced erythema.

### 3.8 PINGs That Regulate Pigmentation

Pigmentation‐based photoprotection strategies typically follow two opposing approaches: either enhancing the production of eumelanin or inhibiting melanogenesis to treat hyperpigmentation disorders. Boosting eumelanin synthesis is increasingly recognized as a proactive defense mechanism against UVR‐induced skin damage, and enhancing its production is thus a promising photoprotection strategy. The best‐studied agent in this category, bergapten (5‐methoxypsoralen; 5‐MOP), has been shown to enhance melanogenesis and significantly reduce DNA damage when used in combination with UVB exposure. Notably, the addition of bergapten to a standard UVB sunscreen conferred sustained photoprotection lasting up to 14 weeks, highlighting its potential as a long‐acting adjunct in sunscreen formulations [110]. However, its mutagenic and carcinogenic nature means it is no longer being exploited for photoprotection. Safer alternatives such as forskolin, which activates cAMP signaling, and salt‐inducible kinase (SIK) inhibitors, which upregulate MITF and pigment gene expression, have shown promise in preclinical studies but lack clinical validation [111, 112, 113].

Excessive sun exposure can also lead to hyperpigmentation, particularly in darker skin types, where it tends to be more persistent and pronounced [114]. Depigmenting agents in sunscreens are increasingly popular, targeting melanin synthesis or its transfer to reduce existing lesions and prevent the formation of new ones. However, few ingredients have been clinically validated to inhibit UVR‐induced pigmentation. Among the best supported are Isobutylamido thiazolyl resorcinol (ITR) [30] and 2‐mercaptonicotinoyl glycine [115]. Inhibiting melanin synthesis in the absence of UV protection, however, could at least theoretically have a negative effect on skin health, and thus the ideal depigmenting ingredient should also have additional photoprotective benefits. Considering this, and according to the data of Brown et al. [7], 18 ingredients are candidates, with the strongest support for p‐coumaric acid (PCA). Seo et al. [31] demonstrated that PCA reduced the pigmentation of human skin whether applied prior to or after UVR exposure. Like ITR, PCA also acts as a tyrosinase inhibitor, but notably, human epidermal melanocytes pre‐treated with PCA exhibited significantly reduced cell death compared to untreated controls [116] following UVR exposure, presumably due to its potent antioxidant activity [117].

While considerable progress has been made in developing photoprotective agents against UVR‐induced pigmentation, the availability of PINGs specifically targeting HEVL‐induced pigmentation remains limited. Lyons et al. [118] reported that topical application of an antioxidant blend comprising diethylhexyl syringylidenemalonate (2%), vitamin E (0.25%), and ascorbyl palmitate (0.01%) was effective at reducing immediate pigmentation 24 h after exposure to blue light + UVA1 in FST IV to VI but had no significant effect on pigmentation measured after 7 days. In line with this observation, topical application of 20% TOC was also reported to have no effect on delayed tanning in skin exposed to VL [119]. Treatment of Normal Human Melanocytes with the antioxidant N‐acetyl‐L‐cysteine (NAC) also did not inhibit melanogenesis following exposure to 415 nm HEVL [56]. Together these data suggest that antioxidants are ineffective at preventing HEVL‐induced neomelanogenesis and, to date, the only clinically effective substances have proven to be iron oxide pigments that scatter blue light before it reaches the skin. Extracts from *Deschampsia antarctica* [120] and *Polypodium leucotomos* [121], however, have shown promise in vitro by inhibiting HEVL‐induced melanin production in melanocytes, though clinical studies are needed to confirm their efficacy.

### 3.9 PINGs That Prevent Photoaging

As photoaging becomes a primary concern for sunscreen users, consumer demand is shifting toward products that not only prevent sunburn but also address the long‐term cellular damage underlying wrinkles and the loss of skin elasticity. Given this trend, it is not surprising that PINGs investigated for their anti‐photoaging benefits represent the largest single category of PINGs identified (Table 1). Brown et al. [7] identified 948 anti‐photoaging ingredients, but the majority (935 ingredients; 98.6%) were supported only by weak or very weak evidence, with only 13 supported by clinical studies. Over half of all listed ingredients (526; 55.5%) were classified as antioxidants. Among these, NAC and TOC emerged as the most substantiated agents [7]. Mechanistic studies further validate their relevance: Kang et al. [32] demonstrated that topical NAC significantly elevated reduced glutathione (GSH) levels in human skin in vivo and inhibited UVR‐induced MMP‐1 expression by blocking AP‐1 signaling [32]. In a related study, NAC (20%) and TOC (5%) were shown to reduce MMP‐12 expression in UVR‐exposed human skin by 54% and 47%, respectively [33]. This is particularly significant because MMP‐12 is the primary enzyme responsible for elastin degradation and is preferentially induced by UVA1 [33, 122, 123, 124]. Since many commercial sunscreens offer incomplete UVA1 protection, the inclusion of effective antioxidants like NAC and TOC may provide a critical compensatory mechanism to prevent UVA1‐mediated ECM breakdown, particularly in deeper dermal layers.

A study by Dong et al. [123] also demonstrated that UVB‐induced DNA damage in keratinocytes leads to the release of soluble mediators, such as cytokines, which in turn stimulate MMP‐1 expression in dermal fibroblasts. Importantly, enhancing DNA repair through the application of photolyase significantly reduced MMP‐1 mRNA and protein levels in human skin. This suggests that CPDs are not only a direct source of mutagenesis but also act as upstream signals for MMP‐mediated ECM degradation. These findings underscore the potential of DNA repair enzymes in mitigating UVR‐induced photoaging by targeting the molecular pathways initiated by CPD formation.

## 4 Discussion

### 4.1 Why Sunscreens Are the Ideal Vehicle for PINGs

Sunscreens offer a uniquely suitable platform for the delivery of PINGs. As daily‐use products designed to remain on the skin during peak exposure periods, sunscreens naturally align with the timing and location of solar radiation‐induced skin damage. Their widespread consumer acceptance, topical nature, and formulation versatility make them an optimal vehicle not only for preventing UV penetration but also for delivering DNA‐repair enzymes, antioxidants, and other bioactive agents that support cellular function. Integrating PINGs into sunscreen formulations enables a dual‐action approach: shielding skin from further harm while actively addressing damage already incurred. Furthermore, this approach offers several distinct advantages over their separate topical application. First, co‐formulation ensures uniform and synergistic photoprotection. When applied together, PINGs are distributed evenly with the UV filters across the skin, maximizing their protective interaction with both sunlight and oxidative stress at the skin's surface. In contrast, separate applications can lead to inconsistent layering and uneven protection.

Second, integration enhances user compliance. Most users prefer simplified routines; combining multiple protective agents into a single product reduces the likelihood of skipping steps and ensures consistent use. This is especially critical in real‐world scenarios, where photoprotection adherence is often suboptimal.

Third, co‐formulating PINGs within sunscreens allows for optimized stability and bioavailability. Formulation scientists can tailor the sunscreen matrix (e.g., emulsifiers, solvents, encapsulation technologies) to stabilize otherwise labile PINGs (like polyphenols or vitamins) and enhance their delivery into the skin. Applied separately, PINGs may degrade faster or fail to penetrate effectively due to incompatibility with the skin environment or preceding products.

Lastly, integrated formulations allow for regulatory and clinical synergy. A single product can be rigorously tested and validated for safety and efficacy, with evidence‐based labeling. Multiple standalone products complicate clinical evaluation and may not provide additive benefits unless specifically studied.

### 4.2 Research Gaps and Future Research Directions

Despite growing interest in PINGs, significant research gaps persist that hinder their systematic incorporation into evidence‐based sunscreen formulations. Among the most pressing challenges is the lack of standardized methodologies for evaluating PING efficacy. Currently, sunscreen performance is typically quantified by the sun protection factor (SPF), which is determined in vivo based on the MED, reflecting the lowest UVR dose required to produce visible skin reddening [59]. However, MED is a limited proxy for biological protection, as it primarily reflects vascular inflammation (erythema) rather than molecular or cellular damage. This metric is particularly ill‐suited for evaluating PINGs, many of which exert their effects through mechanisms such as DNA repair, antioxidant defense, immunoprotection, or pigmentation modulation rather than by filtering UVR.

Critically, MED‐based assessments fail to capture sub‐erythemal or oxidative stress‐related damage and do not account for photodamage induced by VL or IR‐A. Furthermore, there is concern that anti‐inflammatory agents that suppress erythema without preventing underlying damage may artificially inflate SPF values [124]. To advance the clinical integration of PINGs, there is an urgent need for validated, mechanism‐specific endpoints, such as biomarkers of DNA damage, oxidative stress, and pigmentation, which complement or replace conventional SPF testing in both preclinical and clinical evaluations. Although alternative metrics such as the immune protection factor [125], mutation protection factor [126], and free radical protection factor [127] have been proposed, none have been universally adopted. A harmonized, mechanism‐based assessment framework is therefore critically needed.

In parallel, standardized testing protocols and regulatory guidelines tailored to PING‐containing sunscreens remain lacking. Existing frameworks, including those from the FDA and European Commission, focus primarily on UV filter efficacy (e.g., SPF, UVA‐PF), and do not encompass outcomes like antioxidant activity or DNA repair enhancement. This regulatory gap constrains innovation and may contribute to consumer misunderstanding. Moreover, product claims involving natural or botanical components frequently lack rigorous scientific substantiation, highlighting the need for alignment between cosmetic, pharmaceutical, and dermatological regulatory standards. Furthermore, although certain PINGs demonstrate clear biological activity, commercial products often make weak or non‐specific claims to avoid classification as drugs. This situation reflects a long‐standing regulatory gap originally highlighted by Kligman through the term “cosmeceutical” to describe agents that straddle cosmetics and pharmaceuticals [128]. The establishment of an intermediate category could provide a framework for more rigorous evaluation, stimulate innovation, and encourage the generation of higher‐quality clinical evidence for PING‐containing formulations.

To date, most studies evaluating PINGs have concentrated on UVB‐ or UVA‐induced endpoints in controlled laboratory settings, with limited exploration of VL and IR‐A effects. Yet these wavelengths represent an important and underaddressed component of solar radiation. PINGs offer enormous potential to protect skin against these wavelengths, particularly as conventional filters offer limited or no protection. At present, only 17 ingredients with purported HEVL protection have been identified, the majority supported solely by in vitro data [7]. Among them, licorice extract, [72], nicotinamide [129], ethyl ascorbyl ether [130], and *Scenedesmus rubescens* extract [129] show some degree of clinical evidence, though none are backed by robust trials.

Red light (625–700 nm) is also known to induce ROS, inhibit collagen synthesis, and impair fibroblast function in vitro [52]. However, resveratrol remains the only PING with demonstrated protective effects against red light–induced oxidative stress [131]. Similarly, research into IR‐A protection is limited; of the 13 identified PINGs with potential activity, only β‐carotene has been evaluated beyond cell culture [65]. Furthermore, most studies examine these wavebands in isolation, despite emerging evidence that simultaneous exposure to multiple wavelengths induces distinct biological effects. Compounding this issue, many efficacy studies use sub‐erythemal UVR doses, which, while useful for probing molecular endpoints without overt tissue damage, do not replicate real‐world exposure conditions. This underscores the need for standardized, environmentally relevant irradiation models that more accurately reflect natural sunlight.

Another key limitation is the lack of understanding regarding the dose–response relationships and threshold concentrations required for clinically meaningful effects. Existing studies frequently employ differing concentrations, formulation vehicles, and application protocols, complicating cross‐study comparisons. While current evidence suggests that PINGs are particularly effective against oxidative stress and may support endogenous DNA repair, thereby helping to prevent both immediate (e.g., erythema, inflammation, immunosuppression) and long‐term outcomes (e.g., photoaging, carcinogenesis), no single PING confers comprehensive protection against the full spectrum of solar‐induced biological effects. As such, multi‐ingredient formulations leveraging synergistic or complementary mechanisms are likely necessary to achieve full spectrum photoprotection. Whether a “universal” PING exists remains unclear, and future studies should prioritize testing of ingredient combinations for additive or synergistic benefits.

The molecular mechanisms underlying PING activity are also incompletely defined. Addressing this gap will require the application of advanced systems biology approaches, including transcriptomics, proteomics, and metabolomics.

The chemical complexity of many PINGs, particularly botanical extracts, presents additional challenges. Their composition can vary substantially depending on the plant species, harvest conditions, extraction methods, and storage practices. This lack of standardization hinders reproducibility, stability, and regulatory approval. Furthermore, the pharmacokinetics and bioavailability of topically applied PINGs remain poorly understood. Few studies have quantified skin penetration, localization within specific layers, or metabolic fate following application.

Sustainable and ethical sourcing of PINGs, particularly botanicals, should be considered a parallel priority given increasing consumer demand for transparency and environmental responsibility. Equally critical is the effective delivery of PINGs to biologically relevant skin compartments. Advanced delivery systems, such as nanoparticles, liposomes, nanoemulsions, and encapsulation technologies, are being explored to enhance stability, penetration, and bioavailability. However, comparative studies directly evaluating these technologies are scarce, limiting our understanding of their real‐world performance.

Individual variability in skin response, driven by factors such as phototype, genetic background, and overall health, further complicates universal recommendations. Personalized approaches, including the development of adaptive or “intelligent” formulations (e.g., ROS‐ or pH‐responsive systems), represent a promising frontier, offering real‐time protection tailored to environmental stressors.

From a clinical perspective, there is a notable lack of long‐term, placebo‐controlled trials assessing PING‐containing sunscreens across endpoints such as photoaging, pigmentation, immunosuppression, and DNA repair. Of the 1750 PINGs identified by Brown et al. [7], fewer than 10% have been evaluated in clinical studies. Only 27 are supported by strong evidence, and just 18 are approved for inclusion in sunscreens (Table 4). Most existing human trials are short‐term, rely on surrogate endpoints (e.g., erythema or pigmentation scores), and lack histological or molecular validation of long‐term efficacy. Moreover, participant populations often lack gender, ethnic, and phototype diversity, limiting generalizability. This is particularly concerning for PINGs targeting VL‐induced pigmentation, which may be more relevant for darker skin tones. Future trials must ensure diverse representation and include stratified analyses to assess differential efficacy.

**TABLE 4:** Top sunscreen‐compatible PINGs and their endpoint‐specific evidence scores.

Ingredient	Class	Oxidative stress	DNA damage	Cell death	Immunosuppression	Inflammation	Erythema	Pigmentation	Photoaging
α‐Tocopherol	Vitamins	+++++	+	++	+	+	+++++	+	+++
L‐ascorbic acid	Vitamins	+++++	+++	++	+	++	++++	−	++
Tocopheryl acetate	Vitamins	+++	+	+++	−	+	+++++	−	+
Epigallocatechin gallate	Catechins	++++	+	++++	+	++++	+++	−	++
Nicotinamide	Vitamins	+	+	+	+++++	+++	+++	−	+
Melatonin	Biogenic Amines	++	++	++	−	+	+++++	−	+
N‐Acetyl‐L‐cysteine	Amines	+	+	+	+	++	‒	−	+++++
Green tea extract	Plant‐derived substances	+	+++	+++	++++	+	++++	−	+++
Resveratrol	Stilbenes	+	+	+	−	+	+++	−	++
β‐carotene	Carotenoids	+++	++	+	−	+	+++	−	+
*Reseda luteola* extract	Plant‐derived substances	+++	+++	‒	−	+++	+++++	−	−
Sulforaphane	Organosulfur compounds	+	+	+	−	+	++++	−	+
*Potentilla erecta* extract	Plant‐derived substances	−	−	+	−	+++	++++	−	−
T4 Endonuclease V	Enzymes	−	++++	+	++	+++	+	−	+
Green tea polyphenols	Plant‐derived substances	+	+++	+++	+++	+	++++	−	+
*Hypericum perforatum* extract	Plant‐derived substances	++++	−	−	−	−	+++	−	−
Photolyase	Enzymes	+	++++	+++	+++	+	+++	−	+
Quercetin	Flavanols	+	++	+	−	+	++	−	++

*Note:* Evidence strength is based on available clinical, in vivo, and in vitro data as interpreted from Brown et al. [7] and supporting literature. +++++ = very strong evidence; ++++ = strong evidence; +++ = moderate evidence; ++ = weak evidence; + = very weak evidence; − = no evidence.

Finally, formulation stability remains a foundational concern, not only within the sunscreen itself but also with respect to maintaining PING activity upon skin application. Addressing these formulation, mechanistic, and regulatory challenges is essential to unlocking the full potential of PINGs as next‐generation agents for safe, effective, and broad‐spectrum photoprotection.

## 5 Conclusions

PINGs offer a promising solution to many limitations of traditional UV filters, with added benefits for skin health and environmental safety. However, several challenges must be addressed to ensure their broader adoption. Rigorous mechanistic elucidation, clinical validation, formulation optimization, and regulatory alignment are urgently needed to realize their full potential. Bridging these gaps will not only enhance the scientific legitimacy of PINGs but will also facilitate the development of next‐generation sunscreens that are effective, inclusive, and aligned with contemporary dermatological and environmental standards.

## Author Contributions

All authors contributed to conceptualization, methodology, writing – review and editing; Anthony Brown contributed to data curation, validation, formal analysis, writing – original draft, visualization.

## Conflicts of Interest

J.K. serves as a consultant to/IUF obtains funding from: AbbVie, Amway, bitop, Blue Lagoon, Evonik, ISDIN, La Roche‐Posay, L'Oreal, Mary Kay, Meitu, Mistine, Mibelle, Shin, Skinceuticals, Stada, Symrise, Vichy. A.B. and C.T. are employees of ISDIN. T.P. has received grants and/or honoraria from AbbVie, ACM Pharma, Almirall, Amgen, Astellas, Beiersdorf, Bristol Myers Squibb, Calypso, Caudalie, Celgene, Galderma, Genzyme/Sanofi, GlaxoSmithKline, Incyte Corporation, ISDIN, ISIS Pharma, Janssen, LEO Pharma, L'OREAL, Eli Lilly, NAOS, Novartis, Pfizer, Roivant, Sun Pharmaceuticals, SVR, Symrise, Takeda, UCB, and VYNE Therapeutics. He is the cofounder of NIKAIA Pharmaceuticals. C.G. has received honoraria from ISDIN, ACTIVEN. Y.G. has acted as a consultant for Almirall, ISDIN, Roche Posay, Abbvie, Lilly, Sanofi, Pierre Fabre and Pfizer. J.P.‐C. is a consultant for ISDIN. G.L. has acted as consultant for: ISDIN, AVITA Medical, Clinuvel, Incyte Corporation, NAOS. S.S. has received grants and/or honoraria from Cantabria, FQM Brasil, ISDIN, L'OREAL, NAOS, Pierre Fabre. H.W.L. has served as investigator for Incyte, La Roche Posay, Pfizer, and PCORI; consultant for ISDIN, Beiersdorf, Ferndale, L'Oréal, Eli Lilly, Zerigo Health, Skinosive, Kenvue; and speaker on general educational session for La Roche‐Posay, Cantabria labs, Pierre Fabre, NAOS, Uriage, Pfizer, ISDIN.

## Data Availability

Data sharing not applicable to this article as no datasets were generated or analyzed during the current study.
